# Supplementary material for: A systems vaccinology resource to develop and test computational models of immunity
Source: bioRxiv. 2023 Aug 29:2023.08.28.555193. Preprint. [Version 1] doi: 10.1101/2023.08.28.555193 (PMC10491180; doi:10.1101/2023.08.28.555193)
Supplement: Supplement 1 [file media-1.pdf]

Supplementary Materials

A systems vaccinology resource to develop and test computational models of immunity

INDEX

Supplementary tables:

S1. List of previously identified Tdap Immune signatures represented as tasks for 1st challenge.

S2. Implemented prediction methods from literature.

S3. List of antibodies

Supplementary figures:

S1. CMI-PB database schema.

S2. A plot of antibody titer data prior to normalization.

S3. A plot of antibody titer data post normalization.

S4. Identification of published methods to predict vaccine responses.

Reference

Supplementary Tables

Table S1: List of previously identified Immune signatures represented as tasks for the first challenge.

	Task Title	Task statement	Signature	Reference
**Antibody level tasks**				
1	IgG-PT_D14	IgG levels against Pertussis Toxin (PT) antigen in plasma on 14 days post booster vaccination	Vaccine-induced immunity for B. pertussis	[1]]
2	IgG-FHA_D14	IgG levels against Filamentous hemagglutinin (FHA) antigen in plasma on 14 days post booster vaccination	Vaccine-induced immunity for *B. pertussis*	[1]]
3	IgG-Pertactin_D14	IgG levels against Pertactin antigen in plasma on 14 days post booster vaccination	Vaccine-induced immunity for *B. pertussis*	Figure 5 in [1]
4	IgG1-PT_D14	IgG1 levels against PT antigen in plasma on 14 days post booster vaccination	Vaccine-induced immunity for *B. pertussis*	Figure 5 in [1]
5	IgG1-FHA_D14	IgG1 levels against FHA antigen in plasma on 14 days post booster vaccination	Vaccine-induced immunity for *B. pertussis*	Figure 5 in [1]
6	IgG4-PT_D14	IgG4 levels against PT antigen in plasma on 14 days post booster vaccination	Acellular Pertussis (aP) vs. whole-cell Pertussis (wP) response	Figure 5 in [1]
7	IgG4-FHA_D14	IgG4 levels against FHA antigen in plasma on 14 days post booster vaccination	aP vs. wP response	Figure 5 in [1]
**Cell frequency tasks**				
8	Plasmablast_D7	Plasmablast cells on day 7 post-booster vaccination	Vaccine-induced immunity for *B. pertussis*	Figure 3 in [1]
9	CD4TCM_D3	CD4 TCM cells on 3 days post booster vaccination	Vaccine-induced immunity for *B. pertussis*	Figure 3 in [1]
10	Monocytes_D3	Monocytes on day 1 post-booster vaccination	Vaccine-induced immunity for *B. pertussis*	Figure 3 in [1]
**Gene expression tasks**				
11	CCL3_D3	CCL3 on day 3 post booster vaccination	aP vs. wP response	Figure 7 in [1]
12	IL6_D3	IL6 on day 3 post-booster vaccination	aP vs. wP response	Figure 7 in [1]
13	NFKBIA_D7	NFKBIA at day 7 post booster vaccination	aP vs. wP response	Figures 6 and 8 in [1]
14	XIST_D14	XIST on day 14 post-booster vaccination	Biological sex-specific marker	[2]

Table S2: Implemented prediction methods for the first challenge. Models 1-24: Supervised methods based on published vaccine response prediction studies. Models 25-43: unsupervised methods based on Multi-omics dimension reduction.

	Model title	Short Description	Method type	Output score	Experimental data	Reference
1	avey_2017_gene_sig	9-gene signature (RAB24, GRB2, DPP3,ACTB, MVP, DPP7, ARPC4, PLEKHB2, and ARRB1)	Classification (Diagonal Linear Discriminant Analysis: DLDA)	geneset signature score	**Experimental data:** PBMC and whole blood gene expression, cell subset frequencies, antibody titers<br><br>**Pathogen:** Influenza virus	Avey et al. 2017 [3]
2	avey_2017_M54	B-cell signaling Blood transcriptome module (BTM; Module M54)	Classification (DLDA)	geneset signature score		
3	avey_2017_M42	Platelet activation (III) (Module M42)	Classification (DLDA)	geneset signature score		
4	avey_2017_M33	Inflammatory response Module (M33)	Classification (DLDA)	geneset signature score		
5	kotliarov_2020_TGSig	10 gene signature (C2orf63, CD101, ENPP1, RETN, SMC1A, ADAM12, EPHB1, PAPSS2, LONP2, C15orf57)	gene signature score	geneset signature score	**Experimental data:** PBMC and whole blood gene expression, cell subset frequencies, cell surface proteins, antibody titers<br><br>**Pathogen:** Influenza virus, Yellow fever virus	Kotliarov et al. 2020 [4]
6	kotliarov_2020_SLE-Sig	Systemic lupus erythematosus (SLE-Sig) gene signature	gene signature score	geneset signature score		
7	kotliarov_2020_IFN-I-DCact	IFN-I-DCact gene signature	gene signature score	geneset signature score		
8	tsang_2014_DLDA_top2	DLDA model (2 cell populations	Classification (DLDA)	Class probabilities	**Experimental data:** PBMC gene expression, cell subset frequencies, antibody titers<br><br>**Pathogen:** Influenza virus	Tsang et al. 2014 [5][24725414]
9	tsang_2014_DLDA_top5	DLDA model (5 cell populations)	Classification (DLDA)	Class probabilities		
10	fourati_2015_BioAge	BioAge	Classification (Naïve Bayes)	Class probabilities	**Experimental data:** Whole blood gene expression, cell subset frequencies, serum proteins, antibody titers<br><br>**Pathogen:** Hepatitis B virus	Fourati et al. 2015 [6]
11	fourati_2015_M1	M1 of BioAge	Classification (Naïve Bayes)	Class probabilities		
12	fourati_2015_M1+M16	M1+M16 of BioAge	Classification (Naïve Bayes)	Class probabilities		

13	fourati_2015_NB	Naïve Bayes classifier (15 DEGs)	Classification (Naïve Bayes)	The ratio between the post-probabilities and the log odds in the logistic model		
14	fourati_2015_LR	Logistic regression (4 cell populations)	Regression (logistic)	The ratio between the post-probabilities and the log odds in the logistic model		
15	furman_2013_age	gene signature scores Age	Regression (Elastic net)	age	**Experimental data:** Whole blood gene expression, cell subset frequencies serum cytokines, antibody titers, hemagglutinin peptides<br><br>**Pathogen:** Influenza virus	Furman et al. 2013 [7]
16	iulio_2021_HBV_transfer_sig	HBV pre-vaccine transfer signature	Classification (Random Forest)	geneset signature score	**Experimental data:** PBMC and whole blood gene expression<br><br>**Pathogen:** Influenza virus, Hepatitis B virus, Mycobacterium tuberculosis	Iulio et al. 2021 [8]
17	iulio_2021_Inf_M_transfer_sig	Influenza M pre-vaccine transfer signature	Classification (Random Forest)	geneset signature score		
18	iulio_2021_Inf_F_transfer_sig	Influenza F pre-vaccine transfer signature	Classification (Random Forest)	geneset signature score		
19	iulio_2021_TB_transfer_sig	TB pre-vaccine transfer signature	Classification (Random Forest)	geneset signature score		
20	fourati_2021_RF	Random Forest model (top 500 varying genes)	Classification (Random Forest)	Class probabilities	**Experimental data:** PBMC and whole blood gene expression, cell surface proteins, cell subset frequencies, antibody titers<br><br>**Pathogen:** Influenza, smallpox, Yellow fever virus, Pneumococcal meningococcal	Fourati et al. 2021 [9]
21	bartholomeus_2018_gene_sig	23 differentially expressed genes	Classification (Random Forest)	Class probabilities	**Experimental data:** Whole blood gene expression, absolute numbers of white blood cells, red blood cells, and platelets, antibody titers<br><br>**Pathogen:** Hepatitis B virus	Bartholomeus et al. 2018 [10][30205979]
22	bartholomeus_2018_NB	Naïve Bayes classifier (first 5 PCs)	Classification (Naïve Bayes)	Class probabilities		
23	qui_2018_gene_sig	55 up- and 15 down-regulated genes	gene signature score	geneset signature score	**Experimental data:** PBMC gene expression, antibody titers<br><br>**Pathogen:** Hepatitis B virus	Qui et al. 2018 [11]
24	franco_2013_gene_sig	49 genes correlated	gene signature score	geneset signature score	**Experimental data:** Whole blood gene expression,	Franco et

		with antibody response			whole-genome genotyping, antibody titers<br><br>**Pathogen:** Influenza virus	al. 2013 [12]
25	jive.elastic_net_cv	Elastic net regression with cross-validation	Regression (Elastic net)		**Experimental data:** PBMC gene expression, cell frequencies, antibody titers	Lock et al. 2013 [13]
26	jive.elastic_net	Elastic net regression	Regression (Elastic net)			
27	jive.lasso_cv	lasso regression with cross-validation	Regression (lasso)			
28	jive.lasso	lasso regression	Regression (lasso)			
29	jive.lr	Linear regression	Regression (linear)			
30	baseline		Regression (lasso)		Baseline tasks, clinical and demographic features	
31	MCIAbasic		Regression (lasso)		**Experimental data:** transcriptome, cell frequencies, antibody levels, cytokines	
32	MCIAplus		Regression (lasso)		Factors from the MCIAbasic model and features from the baseline model	

Table S3. Antibodies details

Target	Conjugate	Host	Target	Clone	Catalog	Vendor	Application	Dilution
IgG	PE	Mouse	Human	JDC-10	9040-09	Southern Biotech	Antibody response	1/50
IgG1	PE	Mouse	Human	HP6001	9054-09	Southern Biotech	Antibody response	1/50
IgG2	PE	Mouse	Human	HP6025	9070-09	Southern Biotech	Antibody response	1/20
IgG3	PE	Mouse	Human	HP6050	9210-09	Southern Biotech	Antibody response	1/10
IgG4	PE	Mouse	Human	HP6025	9200-09	Southern Biotech	Antibody response	1/10
IgE	PE	Mouse	Human	BE5	MA1-10 375	Thermo Fisher	Antibody response	1/10
CD45	89Y		Human	HI30	308900 3B	Fluidigm	CyTOF	
CD3	115In		Human	UCHT1	300443	Biolegend	CyTOF	
CD19	142Nd		Human	HIB19	314200 1B	Fluidigm	CyTOF	
CD38	144Nd		Human	HIT2	314401 4B	Fluidigm	CyTOF	
CD4	145Nd		Human	RPA-T4	314500 1B	Fluidigm	CyTOF	
CD20	145Nd		Human	2H7	302302	Biolegend	CyTOF	
CD12 3	151Eu		Human	6H6	315100 1B	Fluidigm	CyTOF	
CD45 RA	155Gd		Human	HI100	315501 1B	Fluidigm	CyTOF	
CD1c	160Gd		Human	L161	331502	Biolegend	CyTOF	
CD33	163Dy		Human	WM53	316302 3B	Fluidigm	CyTOF	

CCR7	167Er		Human	G043H7	3167009A	Fluidigm	CyTOF	
CD25	169Tm		Human	M-A251	3169003B	Fluidigm	CyTOF	
CD8a	172Yb		Human	RPA-T8	301002	Biolegend	CyTOF	
CD14	173Yb		Human	61D3	14-0149-82	Thermo Fisher	CyTOF	
HLA-DR	174Yb		Human	L243	3174001B	Fluidigm	CyTOF	
CD56	176Yb		Human	CMSSB	3176003B	Fluidigm	CyTOF	
CD16	209Bi		Human	3G8	3209002B	Fluidigm	CyTOF	

Supplementary Figures

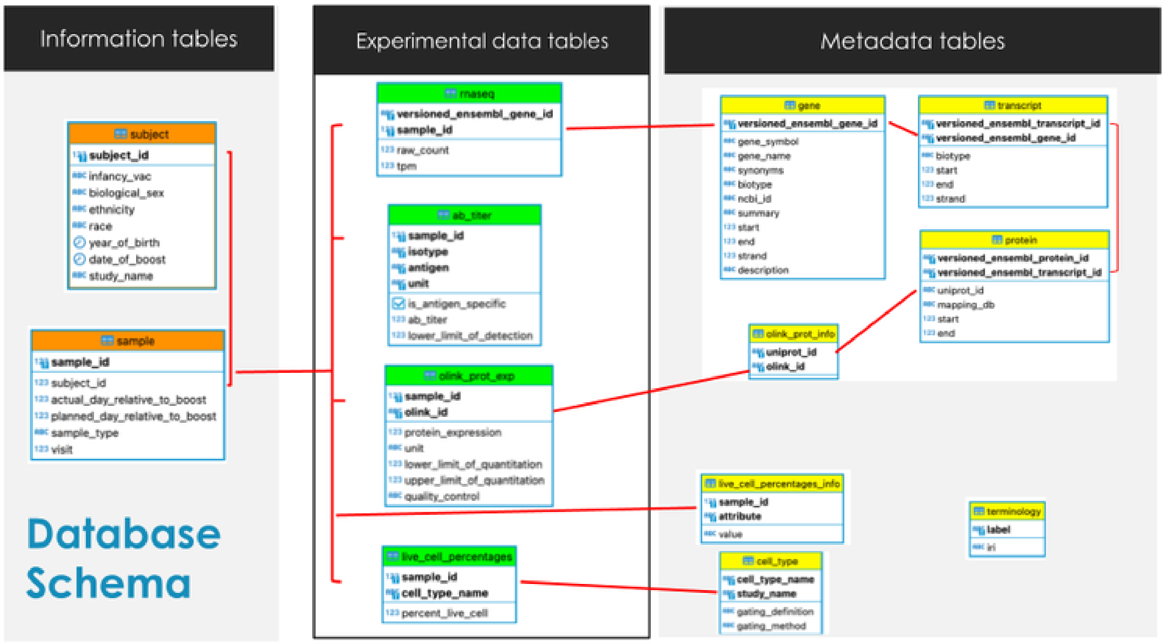

Figure S1: CMI-PB central database schema. The database schema is divided into information, experimental data, and metadata tables. The information tables capture subject and sample information, while the experimental data tables capture experimental data for each omics. In the *rnaseq* table, each row represents an Ensembl gene identifier (versioned_ensembl_gene_id) along with specimen id (specimen_id) and values for measured analytes (raw_count and tpm_count). The *ab_titer* table contains a row for each specimen (specimen_id), with the names and values for measured analytes (isotype, is_antigen_specific, antigen, ab_titer, unit, and lower_limit_of_detection) for the antibody titer experiment. The *olink* table contains a row for each specimen (specimen_id), with the names and values for measured analytes (olink_id and protein_expression). Each row in the *live_cell_percentages* table represents a specimen (specimen_id) along with the names and values for measured analytes (cell_type_name and percent_live_cell). Information on the mapping between olink_id and uniprot_id can be extracted using the *olink_prot_info* table. The metadata tables capture information about ID mapping between CMI-PB data and external databases. The gene, transcript, and protein tables map Ensembl gene, transcript, and protein IDs. These Ensembl IDs are then mapped to UniProt IDs. The *live_cell_percentages_info* and *cell_type* tables provide information about gating information and the experimental technique used to run cell frequency experiments.

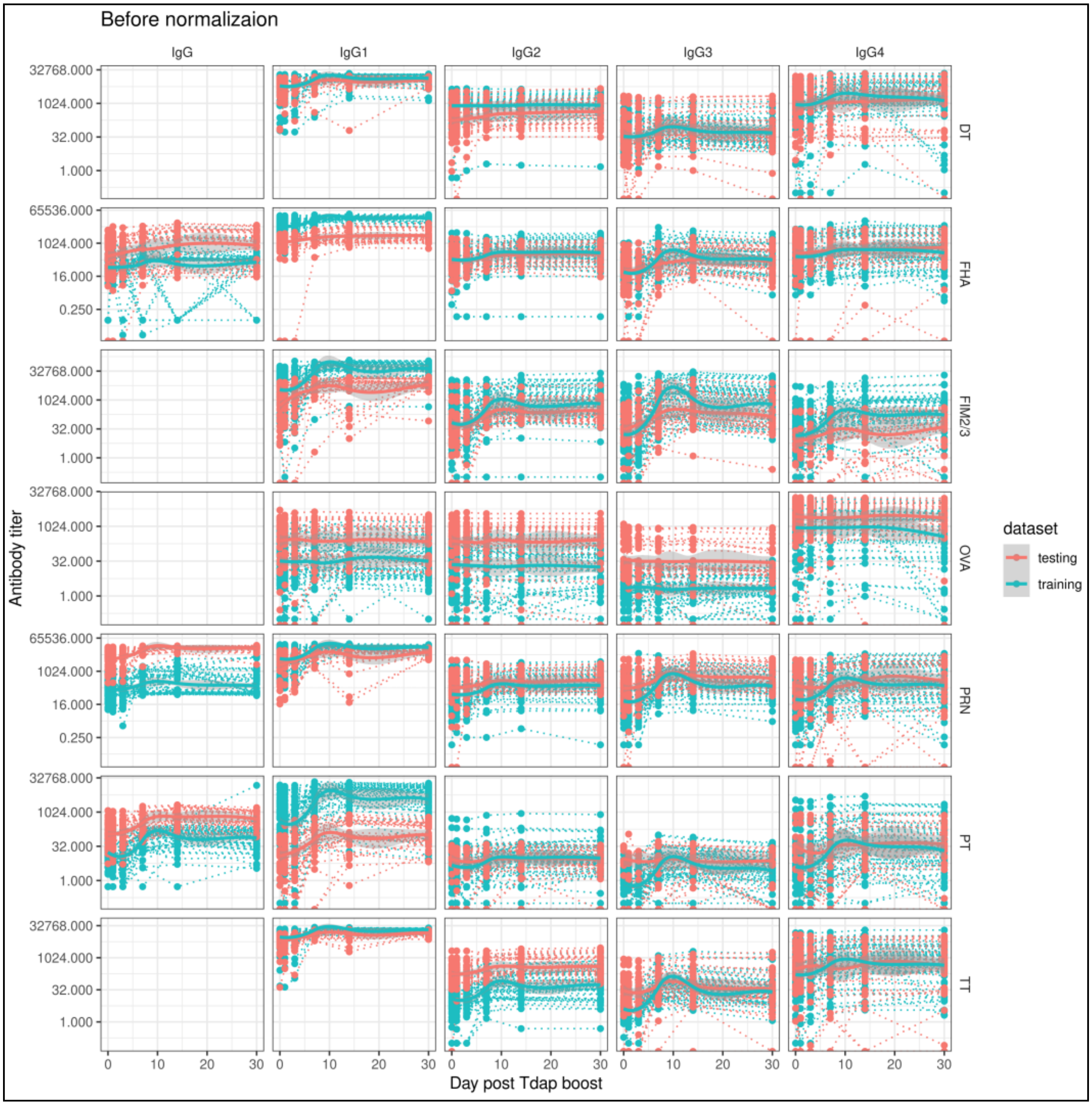

Figure S2: Plot of antibody titer data prior to normalization. Individual plots show the pre- and post-immune response of IgG and its subtypes against antigens. The average titer values differed between the train and test datasets.

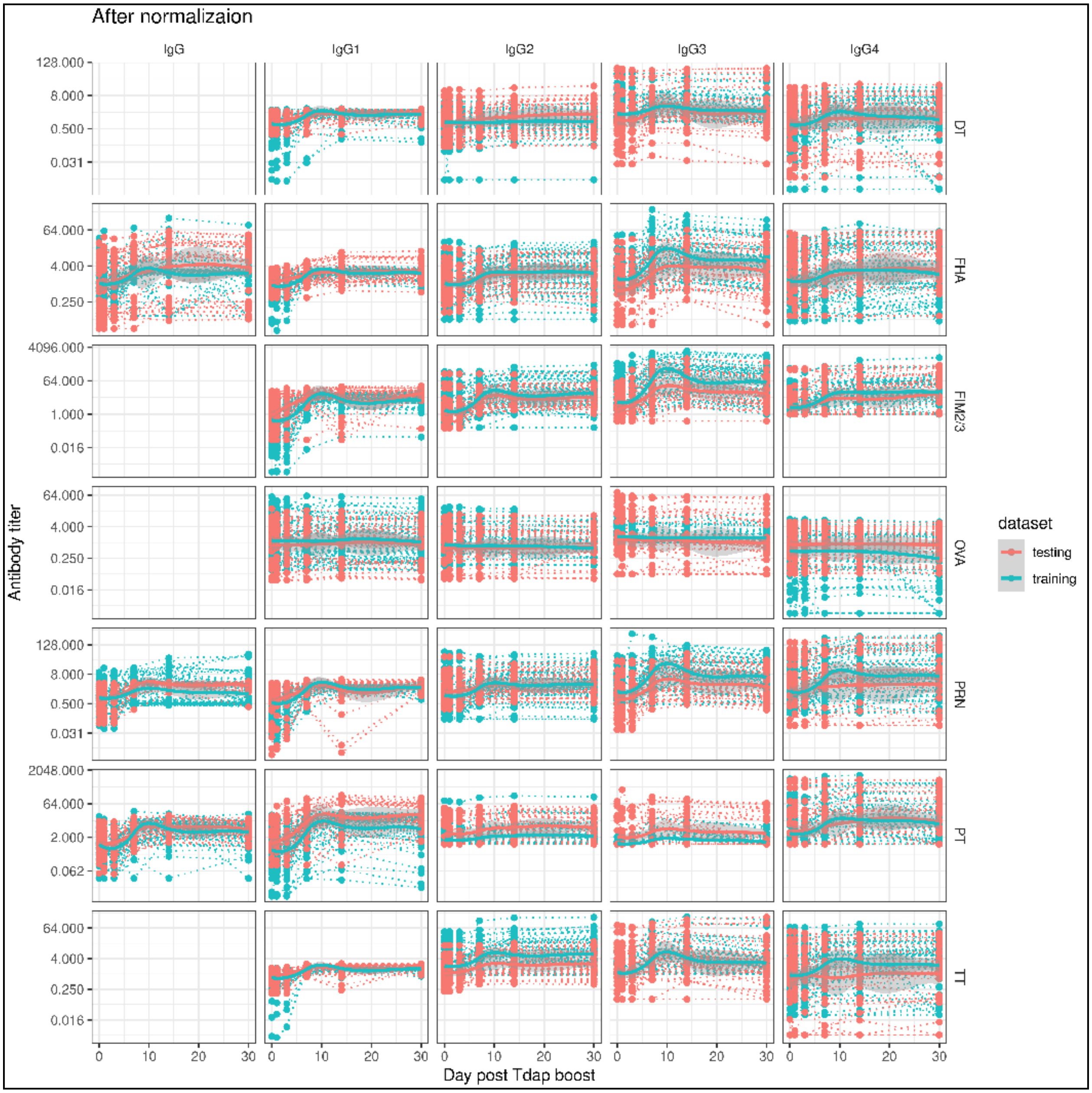

Figure S3: Plot of antibody titer data after normalization. We performed separate standardization of the antibody data in the train and test datasets, using the baseline median as the normalization factor. This approach allowed more direct comparisons of the normalized datasets between the train and test datasets.

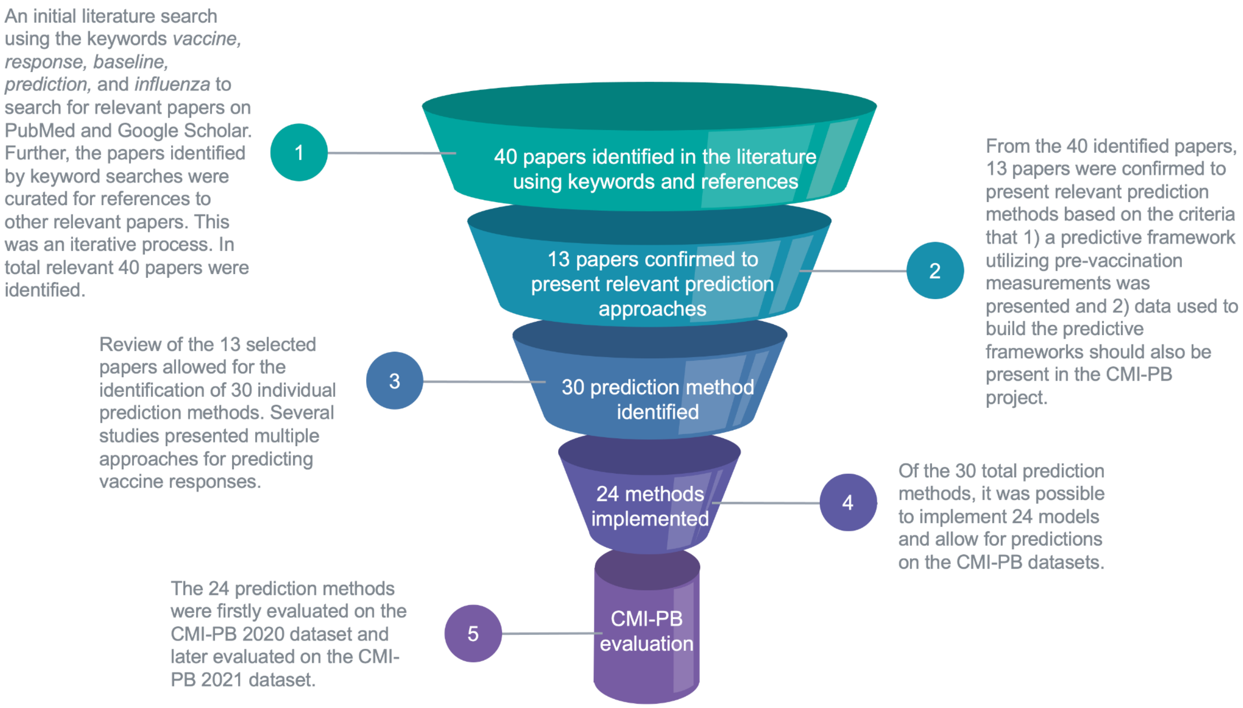

Figure S4: Identification of published methods to predict vaccine responses. The literature search identified previously presented methods for predicting vaccine responses. First, 40 papers were selected from the literature search using keyword searches and relevant references in other papers. From the 40 papers, 13 studies were confirmed to present relevant prediction methods based on a literature search for published vaccine response prediction methods. For several of the 13 relevant studies, multiple prediction methods were presented. In total, 24 prediction methods were implemented from 10 relevant 13 studies (mentioned in **Table S1**). The prediction methods that obtained significant results for both performance metrics in the CMI-PB train datasets were also evaluated in the CMI-PB test dataset used for the first challenge.

References:

1. da Silva Antunes, R. *et al.* A system-view of Bordetella pertussis booster vaccine responses in adults primed with whole-cell versus acellular vaccine in infancy. *JCI Insight* **6**, (2021).
2. Gayen, S., Maclary, E., Hinten, M. & Kalantry, S. Sex-specific silencing of X-linked genes by Xist RNA. *Proc. Natl. Acad. Sci. U. S. A.* **113**, E309-318 (2016).
3. HIPC-CHI Signatures Project Team & HIPC-I Consortium. Multicohort analysis reveals baseline transcriptional predictors of influenza vaccination responses. *Sci. Immunol.* **2**, eaal4656 (2017).
4. Kotliarov, Y. *et al.* Broad immune activation underlies shared set point signatures for vaccine responsiveness in healthy individuals and disease activity in patients with lupus. *Nat Med* **26**, 618–629 (2020).
5. Tsang, J. S. *et al.* Global analyses of human immune variation reveal baseline predictors of postvaccination responses. *Cell* **157**, 499–513 (2014).
6. Fourati, S. *et al.* Pre-vaccination inflammation and B-cell signalling predict age-related hyporesponse to hepatitis B vaccination. *Nat Commun* **7**, 10369 (2016).
7. Furman, D. *et al.* Apoptosis and other immune biomarkers predict influenza vaccine responsiveness. *Mol Syst Biol* **9**, 659 (2013).
8. di Iulio, J., Bartha, I., Spreafico, R., Virgin, H. W. & Telenti, A. Transfer transcriptomic signatures for infectious diseases. *Proc Natl Acad Sci U A* **118**, (2021).
9. Fourati, S. *et al.* Pan-vaccine analysis reveals innate immune endotypes predictive of antibody responses to vaccination. *Nat Immunol* **23**, 1777–1787 (2022).
10. Bartholomeus, E. *et al.* Transcriptome profiling in blood before and after hepatitis B vaccination shows significant differences in gene expression between responders and non-responders. *Vaccine* **36**, 6282–6289 (2018).
11. Qiu, S. *et al.* Significant transcriptome and cytokine changes in hepatitis B vaccine non-responders revealed by genome-wide comparative analysis. *Hum Vaccin Immunother*

14, 1763–1772 (2018).

12. Franco, L. M. *et al.* Integrative genomic analysis of the human immune response to influenza vaccination. *Elife* **2**, e00299 (2013).
13. Lock, E. F., Hoadley, K. A., Marron, J. S. & Nobel, A. B. JOINT AND INDIVIDUAL VARIATION EXPLAINED (JIVE) FOR INTEGRATED ANALYSIS OF MULTIPLE DATA TYPES. *Ann Appl Stat* **7**, 523–542 (2013).
